# Microglia‐containing cerebral organoids model APOE4‐driven Alzheimer's disease pathologies

**DOI:** 10.1002/alz70855_098410

**Published:** 2025-12-23

**Authors:** Daphne Quang, Breanna Dooling, Rose Summers, Huntington Potter, Noah R Johnson

**Affiliations:** ^1^ Department of Neurology, University of Colorado Alzheimer's and Cognition Center, and the Linda Crnic Institute for Down Syndrome, University of Colorado, Anschutz Medical Campus, Aurora, CO, USA

## Abstract

**Background:**

The APOE ε4 allele (APOE4) is the strongest genetic risk factor for Alzheimer's disease (AD) in the typical population and increases the risk for AD in people with Down syndrome (DS‐AD) in comparison to the most common allele, APOE3. Data support the hypothesis that microglial‐apoE interactions drive neuroinflammation and contribute to DS‐AD progression, highlighting a valuable potential therapeutic target for improving cognition and longevity in people with DS‐AD. However, little is known about the mechanisms underlying microglial‐apoE interactions and their contributions to neuroinflammation and neurodegeneration.

**Method:**

We developed a platform using human induced pluripotent stem cell (hiPSC)‐based, microglia‐containing cerebral organoids (MCOs) to: i) gain mechanistic insights into the contributions of APOE4 to DS‐AD, and ii) determine whether novel drugs identified in our screen for inhibitors of the apoE4‐amyloid‐beta (Aβ) interaction prevent or delay DS‐AD phenotypes. We used CRISPR‐Cas9 to convert the genotype from APOE3/3 to APOE4/4 in an hiPSC line derived from a donor with DS, and in an isogenic control hiPSC line disomic for chromosome 21. We then developed MCOs and cerebral organoids without microglia (COs) to investigate how APOE4 affects the microglial contribution to AD pathologies in the DS brain. We also tested compounds from the Spectrum Collection drug library that is comprised of 50% FDA‐approved drugs, 30% natural compounds, and 20% novel small molecule drugs.

**Result:**

In our DS‐AD models, we found that the microglia present in the MCOs, which were not present in the COs, expressed markers of microglial maturity, colocalized with amyloid plaques, and modulated plaque deposition and morphology at different stages of disease. APOE4/4 MCOs exhibited neurodevelopmental and/or neurodegenerative phenotypes resulting in reduced organoid size relative to APOE3/3 MCOs. For the drug screen, our initial exploratory screen of 2,560 compounds identified 23 hit compounds that decreased apoE4‐catalyzed Aβ fibrillization in a ThT‐based amyloid assay. Three of the 23 lead drug candidates reduced intracellular Aβ neuropathology in iPSC‐derived DS neurons, and we will evaluate them using our MCO models of DS‐AD.

**Conclusion:**

MCOs can be utilized to study molecular pathways linking APOE4 to DS‐AD and compounds were discovered to potentially prevent or delay the onset of DS‐AD.

